# Synthesis and Cytotoxicity Evaluation of Tropinone Derivatives

**DOI:** 10.1007/s13659-017-0124-z

**Published:** 2017-03-20

**Authors:** Xiu-Juan Yin, Chang-An Geng, Xing-Long Chen, Chang-Li Sun, Tong-Hua Yang, Tian-Ze Li, Jun Zhou, Xue-Mei Zhang, Ji-Jun Chen

**Affiliations:** 1 State Key Laboratory of Phytochemistry and Plant Resources in West China, Kunming Institute of Botany, Chinese Academy of Sciences, Kunming, 650201 China; 2 Yunnan Key Laboratory of Natural Medicinal Chemistry, Kunming Institute of Botany, Chinese Academy of Sciences, Kunming, 650201 China; 3 University of Chinese Academy of Sciences, Beijing, 100049 China

**Keywords:** Tropinone, Claisen-Schmidt reaction, MTS, Cytotoxicity

## Abstract

**Abstract:**

Sixteen tropinone derivatives were prepared, and their antitumor activities against five human cancer cells (HL-60, A-549, SMMC-7721, MCF-7 and SW480) were evaluated with MTS [3-(4,5-dimethylthiazol-2-yl)-5-(3-carboxy methoxyphenyl)-2-(4-sulfopheny)-2H-tetrazolium] assay. Most of the derivatives exhibited better activities compared with tropinone at the concentration of 40 μM. Particularly, derivative **6** showed significant activities with IC_50_ values of 3.39, 13.59, 6.65, 13.09 and 12.38 μM respectively against HL-60, A-549, SMMC-7721, MCF-7 and SW480 cells, which suggested more potent activities than that of *cis*-dichlorodiamineplatinum (DDP).

**Graphical Abstract:**

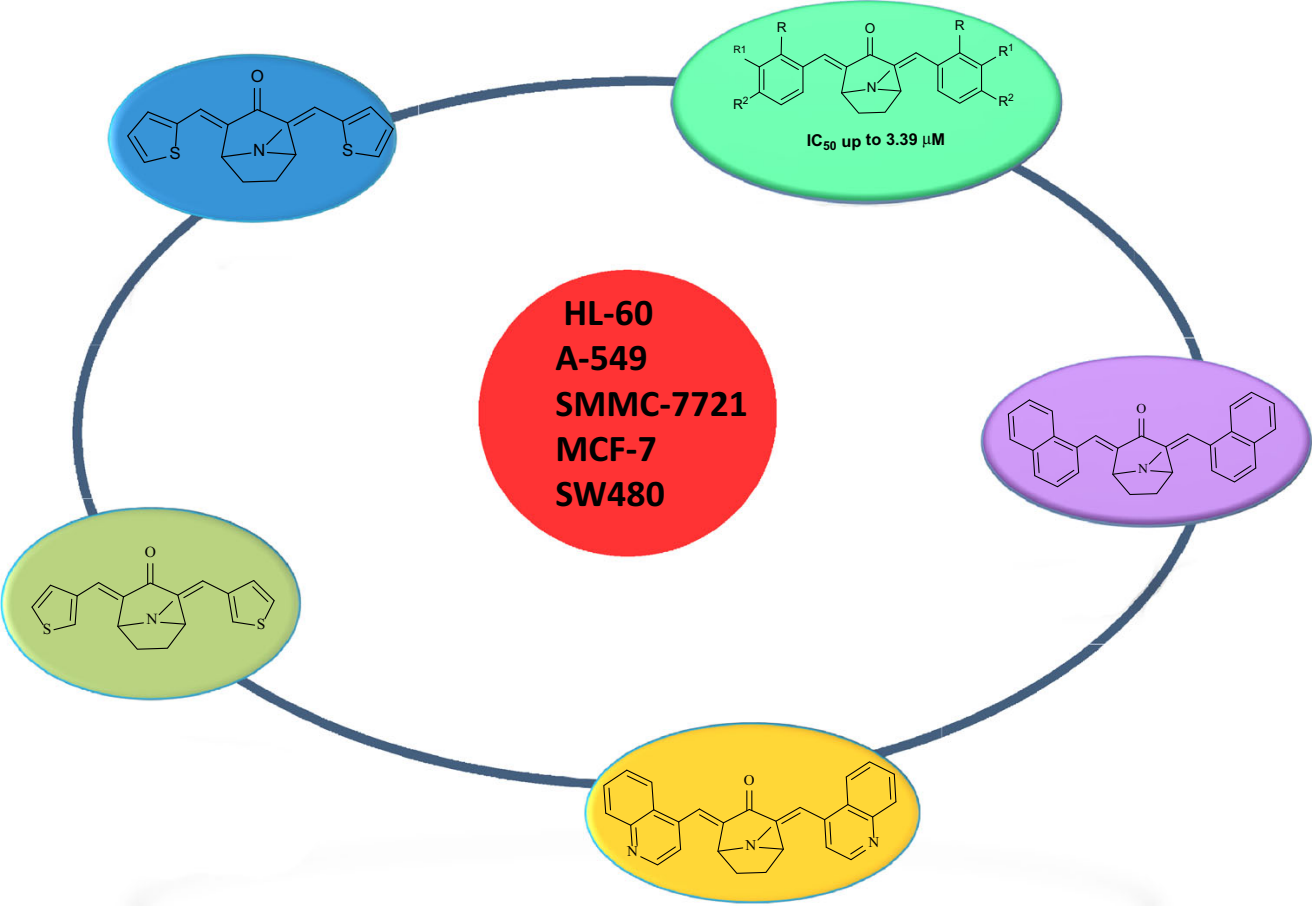

## Introduction

Cancer, a diverse group of diseases characterized by those uncontrolled growth of tumor cells, is a leading cause of morbidity and mortality globally, which brings heavy economic burden to society and individuals [1, 2]. The conquest of cancer continued to pose great challenges to medical science since the pathogenesis is complicated and yet not well clarified [3–5]. With the understanding of cancer pathophysiology, some breakthrough therapies for the treatment of cancer were developed [6], but exploring novel types of antitumor drug is still needed.

Among the natural products studied in the 19th and early 20th centuries, tropane alkaloids attracted particular interest due to their potent and extensive biological activities [7], including regulating the secretion of monoamine neurotransmitter [8–11], glycine receptor [12], and acetylcholine receptor [13–15]. The structural scaffold of tropane is a bicyclic amine with a pyrrolidine and piperidine ring sharing a nitrogen atom and two carbon atoms. Tropinone as a natural tropane alkaloid mainly distributed in Solanaceae plants (*Cyphomandra betacea*) [16], and the total synthesis of tropinone in 1917 by Sir Robert Robinson represented a landmark achievement in organic synthesis [17]. *α,β*-Unsaturated ketone is a kind of important organic intermediate, which is widely used in the fields of medicine, chemistry, material science, biology and so on. Recent studies suggested that chalcones [18–20] and *α,β* unsaturated ketones (including coumarin [21], pyrimidine [22], thiosemicarbazide [23], imidazole [24], piperidine [25] analogues) had antitumor activities. Therefore, it is speculated that *α,β* unsaturated ketones with the structural scaffold of tropane may have antitumor activities. Thus we designed and synthesized a series of tropinone derivatives, and their preliminary biological evaluation was performed for their inhibitory activities in five human cancer cell lines (HL-60, A-549, SMMC-7721, MCF-7 and SW480) using MTS [3-(4, 5-dimethylthiazol-2-yl)-5-(3-carboxymethoxyphenyl)-2-(4-sulfopheny)-2H-tetrazolium] method. The pattern of apoptosis in vitro against cancer depended upon cell line and dose of the compound [26, 27] and the dose was established according to the related literatures [28–30].

## Results and Discussion

### Chemistry

**Fig. 1:**
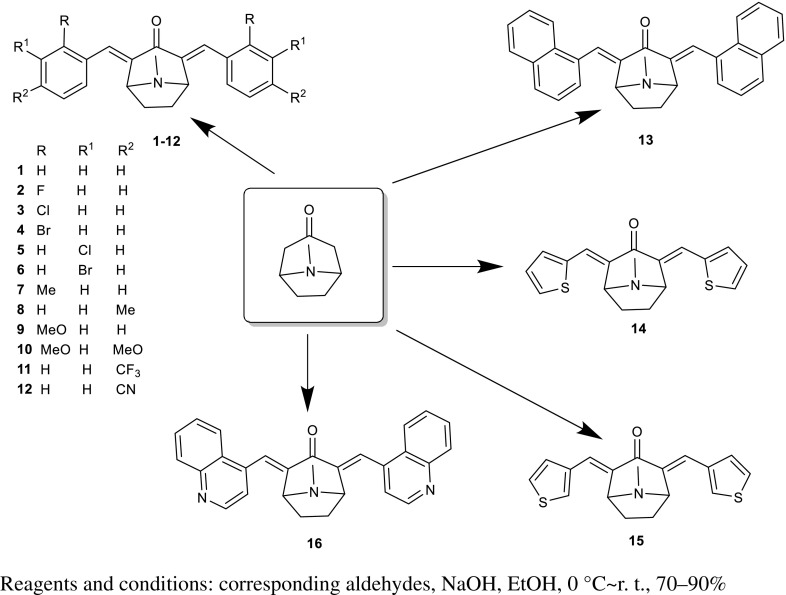
Synthesis of the tropinone derivatives

With an objective to obtain target compounds which have activities against five human cancer cells in vitro, tropinone was reacted with corresponding benzaldehyde or other aromatic aldehydes by Claisen-Schmidt condensation in the mixed solution of a catalytic amount of NaOH, and ethanol to generate compounds **1–16.** The results suggested that introduction of 2,4-*bis*(phenylmethylene) groups improved activities compared with that of tropinone (Fig. 1).

### Biological Evaluation

Derivatives **2** and **8** were synthesized [31–34] and evaluated against Molt 4/C8, CEM cells and L1210 cells in vitro, which were effective indicators of derivatives having potential clinical utility. Derivatives **1–16** were tested for antitumor activities against five human cancer cells (HL-60, A-549, SMMC-7721, MCF-7 and SW480) by MTS method (Tables 1, 2).

**Table 1:** The inhibitory rates of tropinone derivatives against tumor cells in vitro

Comp.	Inhibitory rates (%)^a^				
	HL-60	A-549	SMMC-7721	MCF-7	SW480
DDP	84.33 ± 0.47	70.07 ± 1.50	63.57 ± 3.12	67.28 ± 1.10	59.00 ± 2.10
Taxol	95.81 ± 0.17	73.37 ± 0.28	90.66 ± 0.56	62.28 ± 0.86	56.03 ± 0.31
Tropinone	20.96 ± 2.97	11.38 ± 1.80	8.65 ± 1.67	26.98 ± 2.94	8.68 ± 2.67
**1**	**88.30** ± 3.33	**91.99** **±** **0.51**	**89.16** **±** **3.56**	69.77 ± 1.70	67.55 ± 0.78
**2**	1.32 ± 2.66	32.07 ± 1.16	10.62 ± 1.72	26.05 ± 0.69	32.23 ± 1.77
**3**	6.51 ± 2.36	15.35 ± 2.60	24.96 ± 0.60	38.99 ± 2.21	3.84 ± 4.60
**4**	6.63 ± 2.35	57.58 ± 2.99	25.17 ± .2	51.95 ± 0.22	67.73 ± 2.01
**5**	55.69 ± 2.59	44.10 ± 3.90	44.23 ± 1.94	42.42 ± 1.75	54.20 ± 1.30
**6**	**93.32** **±** **1.22**	**95.17** **±** **0.50**	**94.55** **±** **0.48**	68.19 ± 1.38	67.90 ± 2.42
**7**	28.99 ± 1.20	13.77 ± 0.19	6.53 ± 1.98	5.33 ± 1.49	0.38 ± 1.64
**8**	8.11 ± 3.06	12.21 ± 2.15	1.37 ± 1.10	1.92 ± 1.48	5.98 ± 0.91
**9**	**81.56** **±** **1.98**	67.17 ± 2.80	57.35 ± 0.40	72.91 ± 0.12	**87.84** **±** **1.86**
**10**	59.19 ± 1.64	55.93 ± 0.96	41.75 ± 2.55	26.05 ± 0.54	27.40 ± 1.46
**11**	12.24 ± 1.59	2.44 ± 3.11	1.83 ± 2.85	0.67 ± 0.81	6.65 ± 2.27
**12**	5.93 ± 2.30	9.24 ± 1.86	14.26 ± 2.84	3.56 ± 3.93	18.62 ± 2.98
**13**	39.43 ± 1.47	1.25 ± 1.35	10.87 ± 2.88	16.89 ± 4.58	15.98 ± 1.84
**14**	71.16 ± 2.43	36.96 ± 0.43	33.60 ± 2.70	44.71 ± 1.90	35.28 ± 3.17
**15**	27.50 ± 2.36	54.41 ± 2.93	47.78 ± 0.55	23.19 ± 1.63	22.71 ± 2.21
**16**	2.33 ± 3.08	36.73 ± 3.16	17.06 ± 0.17	60.74 ± 2.60	68.09 ± 1.12

Taxol was tested at the concentration of 5 μM and other derivatives were tested at the concentration of 40 μM

^a^The inhibitory rates expressed as ${\bar{\text{X}}}$  ± SD (*n* = 3)

The derivatives of the significance [bold] showed significant activities against HL-60, A-549, SMMC-7721, MCF-7 and SW480 cells

**Table 2:** The IC_50_ (μM) values of derivatives **1**, **6** and **9** against tumor cells in vitro

Comp.	IC_50_ (μM)				
	HL-60	A-549	SMMC-7721	MCF-7	SW480
DDP	4.31	17.39	13.86	16.31	19.07
Taxol	<0.008	<0.008	<0.008	<0.008	<0.008
**1**	13.62	16.78	14.24	16.57	11.95
**6**	**3.39**	13.59	**6.65**	13.09	12.38
	18.97	29.23	28.90	21.14	19.79

Dose–response of antitumor activity was performed in triplicate and monitored with Thermo Scientific Multiskan FC. IC_50_ values for the derivatives **1**, **6**, **9** and DDP were determined from the dose–response curves obtained with five concentrations from the range of 0.064 to 40 μM against five human cancer cells (HL-60, A-549, SMMC-7721, MCF-7 and SW480), and calculated by the Reed and Muench method [36]. IC_50_ values for taxol were determined from the dose–response curves obtained with five concentrations from the range of 0.008 to 5 μM

The derivatives of the significance [bold] showed significant activities against HL-60, A-549, SMMC-7721, MCF-7 and SW480 cells

Derivative **1** displayed potential inhibitory activity against HL-60 cell, which was very similar with that of derivative **9**, and their inhibitory rates were up to 88.30 ± 3.33% and 81.56 ± 1.98% at the concentration of 40 μM, respectively. Derivatives **1** and **9** with unsubstituted and *ortho*- methoxyl substituted patterns at the phenyl ring, displayed inhibitory potency against HL-60 cell with the IC_50_ values of 13.62 and 18.97 μM, respectively. Compared with the tropinone, the inhibitory activities of derivative **6 (**IC_50_ **=** 3.39 μM) showed a 3–9 fold enhancement and was better than that of the positive DDP. Derivatives **5**, **10** and **14** possessed higher inhibitory activities than that of tropinone with the inhibitory rates of 55.69 ± 2.59, 59.19 ± 1.64 and 71.16 ± 2.43% at the concentration of 40 μM, respectively.

Derivatives **1** and **6** showed significantly inhibitory activities against A-549 cell with IC_50_ values of 16.78 and 13.59 μM, respectively. Derivatives **4**, **9**, **10** and **15** possessed moderate activities with inhibitory rates of 57.58 ± 2.99, 67.17 ± 2.80, 55.93 ± 0.96 and 54.41 ± 2.93% at the concentration of 40 μM, respectively.

Derivatives **1** and **9** displayed inhibitory potency against SMMC-7721 cell with IC_50_ values of 14.24 and 28.90 μM, respectively. Derivative **6 (**IC_50_ **=** 6.65 μM) showed significant activity against SMMC-7721 cell, which exhibited more potential than that of DDP **(**IC_50_ **=** 13.86 μM).

Derivatives **1**, **4**, **6**, **9** and **16** possessed activities against MCF-7 cell with inhibitory rates of 69.77 ± 1.70, 51.95 ± 0.22, 68.19 ± 1.38, 72.91 ± 0.12 and 60.74 ± 2.60% at the concentration of 40 μM, respectively. Particularly, Derivatives **1**, **6** and **9** showed significant activities with IC_50_ values of 16.57, 13.09 and 16.31 μM, respectively.

Derivatives **4**, **5**, **9** and **16** demonstrated moderate inhibitory activity against SW480 cell with inhibitory rates of 67.73 ± 2.01, 54.20 ± 1.30, 87.84 ± 1.86% and 68.09 ± 1.12% at the concentration of 40 μM, respectively.

#### The Preliminary Structure–Activity Relationships (SARs)

**Fig. 2:**
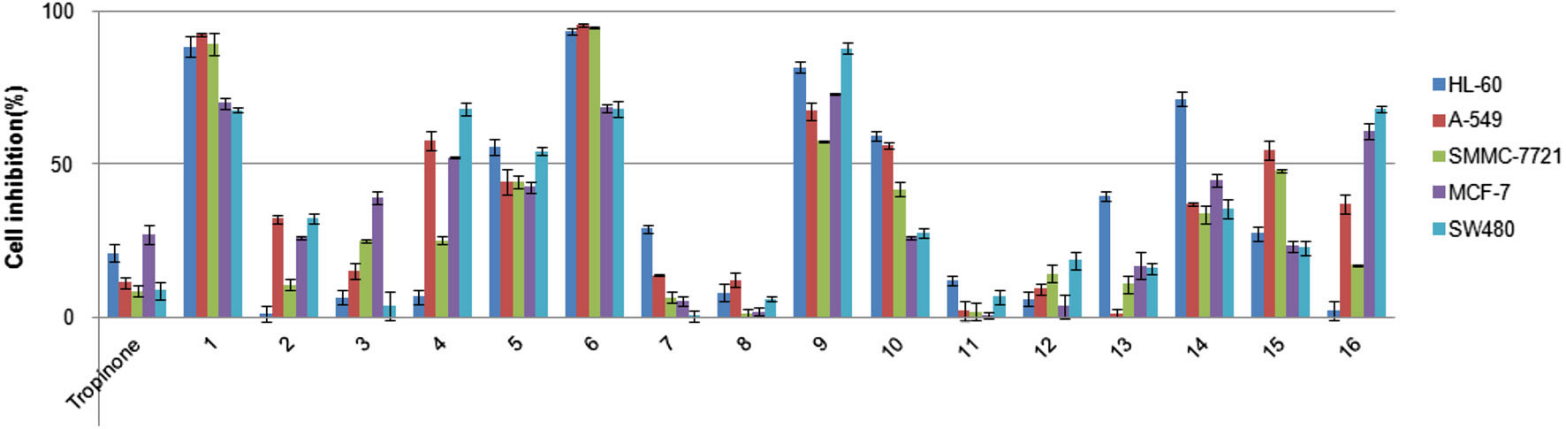
The inhibitory activities of the tropinone derivatives

SARs were discussed based on the bioassay results against five human cancer cells in vitro (Fig. 2). Derivative **1**, with unsubstituted phenylmethylene group, exhibited better activities against five human cancer cells (IC_50_ = 13.62, 16.78, 14.24, 16.57 and 11.95, respectively). Derivatives **2**, **3**, **4** and **7** showed less potent activities, suggesting that the 2-halogenated or 2-methylation derivatives were unfavorable for maintaining activities. The chlorinated derivative **5** at meta-position of phenyl ring displayed slightly lower activities than that of bromated derivative **6** against five human cancer cells. Derivative **6** showed significant activities against HL-60, A-549, SMMC-7721, MCF-7 and SW480 cell (IC_50_ = 3.39, 13.59, 6.65, 13.09 and 12.38, respectively) and more potent activities than that of DDP. Derivative **9** with a methoxy group at the C-2 position of the phenyl ring indicated higher activities than that of dimethoxy substituted derivative **10** with 2,4-position at the phenyl ring. Inhibitory activities of derivatives **8**, **11** and **12**, with electron-donating group (–CH_3_) or an electron-acceptor groups (CF_3_, CN) at the *para*- phenyl ring, showed similar activity against five human cancer cells compared with that of tropinone. From the above results, it is suggested that 2,4-*bis*-phenylmethylene groups were favorable for inhibitory activities. When the 2,4-*bis*-phenylmethylene groups were changed to be 2,4-*bis*-4-heteroaryl-methylene groups, the inhibitory activities significantly decreased (derivatives **14-16** compared to **1-12**), while 2,4-*bis*-naphthylmethylene groups (derivative **13**) did not affect the inhibitory activities.

### Conclusion

In summary, sixteen tropinone derivatives were synthesized and evaluated on HL-60, A-549, SMMC-7721, MCF-7 and SW480 cell lines in vitro. Among of them, derivatives (**1**, **4**, **5**, **6**, **9**, **10**, **14**, **15** and **16**) exhibited higher cytotoxic activities. Particularly, derivatives **1**, **6** and **9** had significantly improved activities against HL-60, A-549, SMMC-7721, MCF-7 and SW480 cells with IC_50_ values of 13.62, 16.78, 14.24, 16.57, 11.95 μΜ, 3.39, 13.59, 6.65, 13.09, 12.38 μΜ and 18.97, 29.23, 28.90, 21.14, 19.79 μΜ respectively. Structurally, the *α*, *β*-unsaturated ketone moiety was introduced into the tropinone scaffold, which should be responsible for enhanced activities. Among the optimized benzene ring derivatives, derivative **6** had higher cytotoxic activities than that of *cis*-DDP. The SARs of tropinone derivatives provided potential compounds for further investigation on antitumor regents screening.

## Experimental Section

### Materials and Instruments

The solvents were dried according to standard procedures. The organic solvents (analytical grade reagents) were purchased from Tianjin Chemical Reagent Co., Ltd (Tianjin, China). The tropinone and corresponding aldehydes were purchased from Alfa Aesar or J&K Scientific Ltd. ^1^HNMR and ^13^CNMR date were recorded in CDCl_3_ on a 400 MHz spectrometer (Bruker, Bremerhaven, Germany) with the tetramethylsilane (TMS) as the internal standard. Low-resolution mass spectra (MS) and high-resolution mass spectra (HRMS) were measured on Shimadzu liquid chromatography-mass spectrometry (LCMS)-ion trap (IT)-time of flight (TOF) (Shimadzu, Kyoto, Japan). All reaction were carried out under an air atmosphere and monitored by using thin-layer chromatography (TLC, 200-300 mesh, Qingdao Makall Group Co., Ltd; Qingdao, China). Melting points which are uncorrected were determined using a SGW^®^ X-4B microscopic melting instrument from Shanghai Precision and Scientific Instrument Co., Ltd (China). The purity of the target compounds was determined by three solvent systems and by HPLC methods.

### General Procedure for the Synthesis of the Tropinone Derivatives

Tropinone (2 mmol, 1 equiv.) and 10% NaOH (5 mL) were dissolved in ethanol (15 mL). To a solution of corresponding aldehydes (4.4 mmol, 2.2 equiv.) in ethanol (5 mL), the mixture was slowly dropped into the above solution at 0 °C for 20 min. The resulting solution was stirred at room temperature and monitored by TLC. Subsequently, the reaction mixture was neutralized with a solution of 5% HCl, extracted with EtOAc (3 × 30 mL) and washed with saturated Na_2_CO_3_ and saturated NaCl. The organic layer was dried over anhydrous Na_2_SO_4_ and concentrated to dryness under reduced pressure. Purification by column chromatography on silica gel Et_2_NH/MeOH/CHCl_3_ (2:6:92, v/v/v) to the target derivatives.

#### 8-Methyl-2,4-bis(phenylmethylene)-8-aza-bicyclo[3.2.1]octan-3-one (**1**)

Yellow powder, mp 137.7–139.6 °C; yield 82%. ^1^H NMR (400 MHz, CDCl_3_) *δ*: 7.84 (s, 2H, H-9, 10), 7.45–7.26 (m, 10H, Ar), 4.40 (m, 2H, H-1, 5), 2.62–2.60 (m, 2H, H-6, 7), 2.30 (s, 3H, H-8), 2.04–2.03 (m, 2H, H-6, 7). ^13^C NMR (100 MHz, CDCl_3_) *δ*: 188.0 (s, C-3), 138.3 (s, C-2,4), 136.6 (d, C-9, 10), 135.1 (s, C-1′, 1″), 130.2 (d, C-4′, 4″), 128.8 (d, C-2′, 2″, 6′, 6″), 128.5 (d, C-3′, 3″, 5′, 5″), 60.8 (d, C-1, 5), 35.8 (s, C-8), 30.3 (t, C-6, 7). IR (KBr) *ν*: 3439, 2950, 1670, 1608, 1584, 1445, 1237, 1164, 946, 778, 692 cm-1. ESIMS: *m/z* 316 [M + H]^+^, HRESIMS: calc for C_22_H_22_NO [M + H]^+^ 316.1696, found 316.1669.

#### 8-Methyl-2,4-bis(2′-fluoro-phenylmethylene)-8-aza-bicyclo[3.2.1]octan-3-one (**2**)

Yellow lamellar crystals (CHCl_3_: EtOH, 80:20, v/v), mp 155.9–156.0 °C; yield 86%, ^1^H NMR (400 MHz, CDCl_3_) *δ*: 7.84 (s, 2H, H-9,10), 7.38–7.10 (m, 8H, Ar–H), 4.22 (m, 2H, H-1, 5), 2.54–2.51 (m, 2H, H-6, 7), 2.30 (s, 3H, H-8), 2.01–1.97 (m, 2H, H-6, 7). ^13^C NMR (100 MHz, CDCl_3_) *δ*: 188.0 (s, C-3), 162.1 (d, C-2′), 159.6 (s, C-2″), 140.2 (s, C-2,4), 130.9 (d, C-9, 10), 130.8 (d, C-4′, 4″), 129.2 (d, C-6′, 6″), 123.9 (s, C-4′, 4″), 123.1 (s, C-1′, 1″), 115.9 (d, C-3′, 3″), 61.1 (d, C-1, 5), 35.4 (s, C-8), 29.9 (t, C-6, 7). IR (KBr) *ν*: 3439, 2943, 1672, 1612, 1589, 1435, 1218, 1056, 752, 685 cm^−1^. ESIMS: *m/z* 352 [M + H]^+^, HRESIMS: calc for C_22_H_19_NOF_2_ [M + H]^+^ 352.1507, found 352.1502.

#### 8-Methyl-2,4-bis(2′-chloro-phenylmethylene)-8-aza-bicyclo[3.2.1]octan-3-one (**3**)

Yellow powder, mp 178.0–178.9 °C; yield 88%, ^1^H NMR (400 MHz, CDCl_3_) *δ*: 7.92 (s, 2H, H-9,10), 7.46–7.19 (m, 8H, Ar–H), 4.14 (m, 2H, H-1, 5), 2.49–2.47 (m, 2H, H-6, 7), 2.31 (s, 3H, H-8), 1.98–1.96 (m, 2H, H-6, 7). ^13^C NMR (100 MHz, CDCl_3_) *δ*: 188.2 (s, C-3), 139.8 (s, C-2, 4), 135.1 (s, C-2′, 2″), 133.7 (d, C-9, 10), 133.7 (s, C-1′, 1″), 130.5 (d, C-3′, 3″), 129.9 (d, C-4′, 4″), 129.8 (d, C-6′, 6″), 126.4 (d, C-5′, 5″), 60.6 (d, C-1, 5), 35.2 (s, C-8), 30.1 (t, C-6, 7). IR (KBr) *ν*: 3438, 2941, 1671, 1610, 1586, 1461, 1218, 1059, 753, 662 cm^−1^. ESIMS: *m/z* 384 [M + H]^+^, HRESIMS: calc for C_22_H_19_NOCl_2_ [M + H]^+^ 384.0916, found 384.0937.

#### 8-Methyl-2,4-bis(2′-bromo-phenylmethylene)-8-aza-bicyclo[3.2.1]octan-3-one (**4**)

Yellow powder, mp 157.4–157.8 °C; yield 87%, ^1^H NMR (400 MHz, CDCl_3_) *δ*: 7.92 (s, 2H, H-9,10), 7.66–7.17 (m, 8H, Ar–H), 4.14 (m, 2H, H-1, 5), 2.48–2.47 (m, 2H, H-6, 7), 2.31 (s, 3H, H-8), 1.98–1.96 (m, 2H, H-6, 7). ^13^C NMR (100 MHz, CDCl_3_) *δ*: 188.2 (s, C-3), 139.7 (s, C-2, 4), 136.0 (d, C-9, 10), 135.5 (s, C-1′, 1″), 135.5 (s, C-2′, 2″), 133.7 (d, C-3′, 3″), 133.0 (d, C-5′, 5″), 129.9 (d, C-6′, 6″), 126.4 (d, C-4′, 4″), 60.6 (d, C-1, 5), 35.1 (s, C-8), 30.1 (t, C-6, 7). IR (KBr) *ν*: 3434, 2936, 1667, 1609, 1558, 1477, 1221, 1062, 942, 786, 688 cm^−1^. ESIMS: *m/z* 471 [M + H]^+^, HRESIMS: calc for C_22_H_19_NOBr_2_ [M + H]^+^ 471.9906, found 471.9928.

#### 8-Methyl-2,4-bis(3′-chloro-phenylmethylene)-8-aza-bicyclo[3.2.1]octan-3-one (**5**)

Yellow powder, mp 136.9–137.8 °C; yield 86%, ^1^H NMR (400 MHz, CDCl_3_) *δ*: 7.71 (s, 2H, H-9,10), 7.34–7.21 (m, 8H, Ar–H), 4.30 (m, 2H, H-1, 5), 2.60–2.58 (m, 2H, H-6, 7), 2.28 (s, 3H, H-8), 2.00–1.96 (m, 2H, H-6, 7). ^13^C NMR (100 MHz, CDCl_3_) *δ*: 187.7 (s, C-3), 142.5 (d, C-9, 10), 139.6 (s, C-2, 4), 137.0 (s, C-3′, 3″), 130.3 (d, C-5′, 5″), 134.9 (d, C-5′, 5″), 129.4 (s, C-2′, 2″), 128.6 (d, C-4′, 4″), 127.1 (d, C-6′, 6″), 61.2 (d, C-1, 5), 36.2 (s, C-8), 30.5 (t, C-6, 7). IR (KBr) *ν*: 3437, 2936, 1667, 1609, 1563, 1477, 1221, 1062, 942, 786, 688 cm^−1^. ESIMS: *m/z* 384 [M + H]^+^, HRESIMS: calc for C_22_H_19_NOCl_2_ [M + H]^+^ 384.0916, found 384.0916.

#### 8-Methyl-2,4-bis(3′-bromo-phenylmethylene)-8-aza-bicyclo[3.2.1]octan-3-one (**6**)

Yellow powder, mp 142.4–143.7 °C; yield 84%, ^1^H NMR (400 MHz, CDCl_3_) *δ*: 7.72 (s, 2H, H-9,10), 7.50–7.26 (m, 8H, Ar–H ‘), 4.32 (m, 2H, H-1, 5), 2.62–2.59 (m, 2H, H-6, 7), 2.30 (s, 3H, H-8), 2.02–1.97 (m, 2H, H-6, 7). ^13^C NMR (100 MHz, CDCl_3_) *δ*: 187.4 (s, C-3), 139.4 (d, C-2, 4), 137.0 (s, C-1′, 1″), 134.9 (d, C-9, 10), 132.9 (d, C-2′, 2″), 131.7 (d, C-4′, 4″), 130.0 (d, C-5′, 5″), 128.6 (d, C-6′, 6″), 122.6 (s, C-3′, 3″), 60.7 (d, C-1, 5), 35.9 (s, C-8), 30.1 (t, C-6, 7). IR (KBr) *ν*: 3435, 2929, 1668, 1608, 1556, 1470, 1220, 1058, 944, 787, 681 cm^−1^. ESIMS: *m/z* 471 [M + H]^+^, HRESIMS: calc for C_22_H_19_NOBr_2_ [M + H]^+^ 471.9906, found 471.9928.

#### 8-Methyl-2,4-bis(2′-methyl-phenylmethylene)-8-aza-bicyclo[3.2.1]octan-3-one (**7**)

Yellow powder, mp 151.0–152.2 °C; yield 79%, ^1^H NMR (400 MHz, CDCl_3_) *δ*: 7.93 (s, 2H, H-9,10), 7.27–7.13 (m, 8H, Ar–H), 4.20 (m, 2H, H-1, 5), 2.51–2.48 (m, 2H, H-6, 7), 2.36 (s, 3H, H-8), 2.29 (s, 6H, Me), 2.00–1.98 (m, 2H, H-6, 7). ^13^C NMR (100 MHz, CDCl_3_) *δ*: 188.8 (s, C-3), 138.8 (s, C-2, 4), 138.0 (s, C-1′, 1″), 135.4 (d, C-9, 10), 134.3 (s, C-2′, 2″), 130.3 (d, C-4′, 4″), 129.2 (d, C-3′, 3″), 128.7 (d, C-6′, 6″), 125.6 (s, C-5′, 5″), 60.8 (d, C-1, 5), 35.6 (s, C-8), 30.3 (t, C-6, 7), 20.2 (s, C_2′, 2″_-Me). IR (KBr) *ν*: 3439, 2944, 1669, 1612, 1594, 1452, 1205, 1058, 925, 778 cm^−1^. ESIMS: *m/z* 344 [M + H]^+^, HRESIMS: calc for C_24_H_25_NO [M + H]^+^ 344.2009, found 344.1993.

#### 8-Methyl-2,4-bis(4′-methyl-phenylmethylene)-8-aza-bicyclo[3.2.1]octan-3-one (**8**)

Yellow powder, mp 165.5–166.5 °C; yield 78%, ^1^H NMR (400 MHz, CDCl_3_) *δ*: 7.83 (s, 2H, H-9,10), 7.54 (d, J = 7.9 Hz, 4H, Ar–H), 7.23 (d, *J* = 7.9 Hz, 4H, Ar–H), 4.40 (m, 2H, H-1, 5), 2.63–2.60 (m, 2H, H-6, 7), 2.38 (s, 3H, H-8), 2.30 (s, 6H, Me), 2.04–1.99 (m, 2H, H-6, 7). ^13^C NMR (100 MHz, CDCl_3_) *δ*: 187.8 (s, C-3), 139.2 (s, C-2, 4), 137.5 (s, C-1′, 1″), 136.7 (d, C-9, 10), 132.2 (s, C-4′, 4″), 130.4 (d, C-3′, 3″, 5′, 5″), 129.3 (d, C-2′, 2″, 6′, 6″), 60.9 (d, C-1, 5), 35.7 (s, C-8), 30.3 (t, C-6, 7), 21.4 (s, C_4′, 4″_-Me). IR (KBr) *ν*: 3438, 2942, 1666, 1599, 1580, 1447, 1239, 1057, 935, 812 cm^−1^. ESIMS: *m/z* 344 [M + H]^+^, HRESIMS: calc for C_24_H_25_NO [M + H]^+^ 344.2009, found 344.1993.

#### 8-Methyl-2,4-bis(2′-methoxyl-phenylmethylene)-8-aza-bicyclo[3.2.1]octan-3-one (**9**)

Yellow lamellar crystals (CHCl_3_: EtOH, 80:20, v/v), mp 155.5–155.7 °C; yield 70%, ^1^H NMR (400 MHz, CDCl_3_) *δ*: 8.02 (s, 2H, H-9,10), 7.35–6.90 (m, 8H, Ar–H), 4.27 (m, 2H, H-1, 5), 3.82 (s, 6H, OMe), 2.53–2.51 (m, 2H, H-6, 7), 2.30 (s, 3H, H-8), 2.02–1.99 (m, 2H, H-6, 7). ^13^C NMR (100 MHz, CDCl_3_) *δ*: 188.5 (s, C-3), 158.4 (s, C-2′, 2″), 138.2 (s, C-2, 4), 132.5 (d, C-9, 10), 130.5 (d, C-4′, 4″), 130.5 (d, C-6′, 6″), 124.3 (s, 1′, 1″), 120.0 (d, C-5′, 5″), 110.7 (d, C-3′, 3″), 61.0 (d, C-1, 5), 55.4 (s, C_2′, 2″_-OMe), 35.2 (s, C-8), 30.3 (t, C-6, 7). IR (KBr) *ν*: 3441, 2944, 1673, 1598, 1486, 1462, 1250, 1058, 757 cm^−1^. ESIMS: *m/z* 376 [M + H]^+^, HRESIMS: calc for C_24_H_25_NO_3_ [M + H]^+^ 376.1907, found 376.1906.

#### 8-Methyl-2,4-bis(2′,4′-dimethoxy-phenylmethylene)-8-aza-bicyclo[3.2.1]octan-3-one (**10**)

Yellow oil, yield 79%,^1^H NMR (400 MHz, CDCl_3_) *δ*: 7.95 (s, 2H, H-9,10), 7.10–6.41 (m, 6H, Ar–H), 4.23 (m, 2H, H-1, 5), 3.78 (s, 12H, OMe), 2.53–2.49 (m, 2H, H-6, 7), 2.34 (s, 3H, H-8), 1.94–1.84 (m, 2H, H-6, 7). ^13^C NMR (100 MHz, CDCl_3_) *δ*: 188.0 (s, C-3), 161.7 (s, C-4′, 4″), 159.8 (s, C-2′, 2″), 136.3 (s, C-2, 4), 132.2 (d, C-9, 10), 131.1 (d, C-6′, 6″), 117.1 (s, C-1′, 1″), 104.1 (s, C-5′, 5″), 98.2 (d, C-3′, 3″), 60.9 (d, C-1, 5), 58.4 (s, C_2′, 2″_-OMe), 55.2 (s, C_4′, 4″_-OMe), 35.0 (s, C-8), 30.3 (t, C-6, 7). IR (KBr) *ν*: 3438.45, 2941.52, 1677.93, 1603.77, 1502.15, 1463.85, 1245.95, 1059.72, 810.43 cm^−1^. ESIMS: *m/z* 436 [M + H]^+^, HRESIMS: calc for C_26_H_29_NO_5_ [M + H]^+^ 436.2118, found 436.2128.

#### 8-Methyl-2,4-bis(4′-trifluoromethyl-phenylmethylene)-8-aza-bicyclo[3.2.1]octan-3-one (**11**)

Yellow lamellar crystals (CHCl_3_: EtOH, 80:20, v/v), mp 172.8–173.2 °C; yield 88%, ^1^H NMR (400 MHz, CDCl_3_) *δ*: 7.81 (s, 2H, H-9,10), 7.68 (d, *J* = 7.8 Hz, 4H, Ar–H), 7.48 (d, J = 7.8 Hz, 4H, Ar–H), 4.33 (m, 2H, H-1, 5), 2.64–2.61 (m, 2H, H-6, 7), 2.30 (s, 3H, H-8), 2.05–2.02 (m, 2H, H-6, 7). ^13^C NMR (100 MHz, CDCl_3_) *δ*: 187.4 (s, C-3), 140.1 (s, C-2, 4), 138.4 (s, C-1′, 1″), 134.8 (d, C-9, 10), 130.3 (d, C-4′, 4″), 130.2 (d, C-3′, 3″, 5′, 5″), 125.5 (d, C-2′, 2″, 6′, 6″), 125.5 (s, C_4′, 4″_-CF_3_), 60.8 (d, C-1, 5), 35.9 (s, C-8), 30.1 (t, C-6, 7). IR (KBr) *ν*: 3440, 2935, 1672, 1609, 1587, 1411, 1244, 1065, 929, 842 cm^−1^. ESIMS: *m/z* 452 [M + H]^+^, HRESIMS: calc for C_24_H_19_NOF_6_ [M + H]^+^ 452.1444, found 452.1450.

#### 8-Methyl-2,4-bis(4′-cyano-phenylmethylene)-8-aza-bicyclo[3.2.1]octan-3-one (**12**)

Yellow powder, mp 249.3–249.8 °C; yield 79%, ^1^H NMR (400 MHz, CDCl_3_) *δ*: 8.08 (s, 2H, H-9,10), 7.81–7.70 (m, 4H, Ar–H), 7.46–7.42 (m, 4H, Ar–H), 4.28 (m, 2H, H-1, 5), 2.63–2.60 (m, 2H, H-6, 7), 2.29 (s, 3H, H-8), 2.01–1.98 (m, 2H, H-6, 7). ^13^C NMR (100 MHz, CDCl_3_) *δ*: 187.1 (s, C-3), 140.7 (s, C-2, 4), 139.4 (s, C-1′, 1″), 134.2 (d, C-9, 10), 132.3 (d, C-3′, 3″, 5′, 5″), 130.5 (d, C-2′, 2″, 6′, 6″), 118.4 (s, C-4′, 4″), 112.3 (s, C_4′, 4″_-CN), 60.8 (d, C-1, 5), 36.0 (s, C-8), 29.9 (t, C-6, 7). IR (KBr) *ν*: 3431, 2942, 2227, 1670, 1605, 1585, 1501, 1241, 1060, 944, 835 cm^−1^. ESIMS: *m/z* 366 [M + H]^+^, HRESIMS: calc for C_24_H_19_N_3_O [M + H]^+^ 366.1601, found 366.1585.

#### 8-Methyl-2,4-bis(2′-naphthylmethylene)-8-aza-bicyclo[3.2.1]octan-3-one (**13**)

Yellow powder, mp 257.6–258.1 °C; yield 78%, ^1^H NMR (400 MHz, CDCl_3_) *δ*: 8.02 (s, 2H, H-9,10), 7.90–7.86 (m, 8H, Ar–H), 7.54–7.52 (m, 6H, naphthalene-H), 4.54–4.53 (m, 2H, H-1, 5), 2.73–2.70 (m, 2H, H-6, 7), 2.48 (s, 3H, H-8), 2.19–2.17 (m, 2H, H-6, 7). ^13^C NMR (100 MHz, CDCl_3_) *δ*: 188.0 (s, C-3), 138.7 (s, C-2, 4), 136.7 (d, C-9, 10), 133.2 (s, C-1′, 1″), 133.1 (s, C-10′, 10″), 132.7 (s, C-9′, 9″), 130.3 (d, C-5′, 5″), 128.5 (d, C-4′, 4″), 128.2 (d, C-3′, 3″), 127.7 (d, C-7′, 7″), 127.4 (d, C-6′, 6″), 127.0 (d, C-8′, 8″), 126.6 (d, C-2′, 2″),61.1 (d, C-1, 5), 35.9 (s, C-8), 30.4 (t, C-6, 7). IR (KBr) *ν*: 3442, 2947, 1670, 1612, 1586, 1440, 1207, 1153, 1056, 940, 822, 747 cm^−1^. ESIMS: *m/z* 416 [M + H]^+^, HRESIMS: calc for C_30_H_25_NO [M + H]^+^ 416.2009, found 416.1996.

#### 8-Methyl-2,4-bis(2′-thienylmethylene)-8-aza-bicyclo[3.2.1]octan-3-one (**14**)

Yellow powder, mp 163.6–164.5 °C; yield 74%, ^1^H NMR (400 MHz, CDCl_3_) *δ*: 7.92 (s, 2H, H-9,10), 7.52–7.11 (m, 6H, thiophene-H), 4.65 (m, 2H, H-1, 5), 2.61–2.58 (m, 2H, H-6, 7), 2.42 (s, 3H, H-8), 1.84–1.79 (m, 2H, H-6, 7). ^13^C NMR (100 MHz, CDCl_3_) *δ*: 186.6 (s, C-3), 138.3 (s, C-2, 4), 135.6 (s, C-2′, 2″), 133. 2 (d, C-9, 10), 130.1 (d, C-5′, 5″), 128.8 (d, C-3′, 3″), 127.9 (d, C-4′, 4″), 61.2 (d, C-1, 5), 36.2 (s, C-8), 29.7 (t, C-6, 7). IR (KBr) *ν*: 3437, 2944, 1655, 1589, 1450, 1417, 1239, 1166, 1040, 932, 853, 706 cm^−1^. ESIMS: *m/z* 328 [M + H]^+^, HRESIMS: calc for C_18_H_17_NOS_2_ [M + H]^+^ 328.0824, found 328.0808.

#### 8-Methyl-2,4-bis(3′-thienylmethylene)-8-aza-bicyclo[3.2.1]octan-3-one (**15**)

Yellow powder, mp 169.9–170.8 °C; yield 77%, ^1^H NMR (400 MHz, CDCl_3_) *δ*: 7.78 (s, 2H, H-9,10), 7.74–7.22 (m, 6H, thiophene-H), 4.48 (m, 2H, H-1, 5), 2.63–2.61 (m, 2H, H-6, 7), 2.38 (s, 3H, H-8), 1.97–1.92 (m, 2H, H-6, 7). ^13^C NMR (100 MHz, CDCl_3_) *δ*: 187.4 (s, C-3), 136.8 (s, C-2, 4), 136.6 (s, C-3′, 3″), 130.1 (d, C-9, 10), 129.1 (d, C- 2′, 2″), 128.3 (d, C- 4′, 4″), 126.2 (d, C- 5′, 5″), 61.2 (d, C-1, 5), 36.0 (s, C-8), 30.0 (t, C-6, 7). IR (KBr) *ν*: 3444, 2934, 1673, 1608, 1582, 1380, 1240, 1204, 1152, 1058, 928, 789 cm^−1^. ESIMS: *m/z* 328 [M + H]^+^, HRESIMS: calc for C_18_H_17_NOS_2_ [M + H]^+^ 328.0824, found 328.0804.

#### 8-Methyl-2,4-bis(2′quinolylmethylene)-8-aza-bicyclo[3.2.1]octan-3-one (**16**)

Yellow powder, mp 177.3–178.0 °C; yield 76%, ^1^H NMR (400 MHz, CDCl_3_) *δ*: 8.87 (d, *J* = 4.3 Hz, 2H, quinoline-H), 8.19 (s, 2H, H-9,10), 8.10–7.92 (m, 4H, quinoline-H), 7.69–7.49 (m, 4H, quinoline-H), 7.10 (d, J = 4.3 Hz, 2H, quinoline-H), 4.04 (m, 2H, H-1, 5), 2.38 (m, 2H, H-6, 7), 2.18 (s, 3H, H-8), 1.93–1.88 (m, 2H, H-6, 7). ^13^C NMR (100 MHz, CDCl_3_) *δ*: 187.3 (s, C-3), 149.6 (d, C-2′, 2″), 148.2 (s, C-4′, 4″), 142.4 (s, C-9′, 9″), 140.7 (s, C-2, 4), 1301.7 (d, C-9, 10), 130.0 (d, C-7′, 7″),129.8 (d, C-8′, 8″), 127.1 (d, C-6′, 6″), 126.7 (s, C-10′, 10″), 124.5 (d, C-5′, 5″), 120.7 (d, C-3′, 3″), 61.0 (d, C-1, 5), 35.820 (s, C-8), 29.9 (t, C-6, 7). IR (KBr) *ν*: 3441, 2943, 1683, 1612, 1580, 1563, 1503, 1417, 1388, 1204, 1166, 1061, 891, 845, 768 cm^−1^. ESIMS: *m/z* 418 [M + H]^+^, HRESIMS: calc for C_28_H_23_N_3_O [M + H]^+^ 418.1914, found 418.1932.

## Biological Test Methods

The human tumor cell lines HL-60, SMMC-7721, A-549, MCF-7 and SW-480 were used, which were obtained from ATCC (Manassas, VA, USA). All cells were cultured in RPMI-1640 or DMEM medium (Hyclone, Logan, UT, USA), supplemented with 10% fetal bovine serum (Hyclone) and cultured with 95% O_2_/5% CO_2_ at 37 °C. Cell viability was assessed by conducting colorimetric measurements of the amount of insoluble formazan formed in living cells based on the reduction of 3-(4,5-dimethylthiazol-2-yl)-5-(3-carboxymethoxyphenyl)-2-(4-sulfopheny)-2H-tetrazolium (MTS, Sigma, St. Louis, MO, USA) [35]. The cells were seeded in a Matrigel coated 96-well black plate with a plating volume of 100 μL/well at a density of 3000–15,000/well, and incubated in CO_2_ incubator to adhere for 12 h before the tested drugs were added. Tested derivatives and positive drug were dissolved in dimethyl sulfoxide (DMSO) and extracted a plating volume of 20 μL/well in the Matrigel coated 96-well clear plate. With cisplatin and paclitaxel as positive controls, each tumor cell line was exposed to the test derivatives at 40 μM concentrations in triplicate for 48 h. After the incubation, MTS (20 μL) was added to each well, and the incubation continued for 2–4 h at 37 °C. The optical density of lysate was measured at 492 nm in a 96-well by Multiskan FC (Thermo Scientific, US). The inhibition rates expressed as ${\bar{\text{X}}}$  ± SD (n = 3) were obtained. The IC_50_ value of each derivative was calculated by Reed and Muench’s method [36].
